# SNW1 promotes lymphatic metastasis in bladder cancer by modulating SRPK1 splicing

**DOI:** 10.1016/j.isci.2026.114811

**Published:** 2026-01-27

**Authors:** Jijie Hu, Ying Zhu, Guoli Wang, Huiqi Dai, Haoyuan Han, Wenmin Cao, Meng Ding, Wenli Diao, Qun Lu, Qing Zhang, Hongqian Guo, Wei Chen

**Affiliations:** 1 Department of Urology, Nanjing Drum Tower Hospital, Clinical College of Nanjing University of Chinese Medicine, Nanjing, China; 2 Department of Urology, Nanjing Drum Tower Hospital, The Affiliated Hospital of Nanjing University Medical School, Institute of Urology, Nanjing University, Nanjing, China; 3 Department of Urology, Joint Institute of Nanjing Drum Tower Hospital for Life and Health, College of Life Science, Nanjing Normal University, Nanjing, China

**Keywords:** biochemistry, cancer, omics

## Abstract

The overall survival of patients with metastatic bladder cancer (BCa) remains poor. There is a lack of effective therapies and reliable biomarkers for metastatic BCa. BCa cells initially disseminate via the pelvic lymph nodes (LN). This study identified that elevated levels of SNW1 have been correlated with LN metastasis and unfavorable patient outcomes. SNW1 enhances the migratory and invasive capabilities of BCa cells. Further investigation revealed that SNW1 modulates SRPK1 splicing, resulting in opposite expression changes in two distinct SRPK1 transcripts. The effect of SNW1 on promoting metastasis is dependent on SRPK1 protein expression. Mechanistic investigations revealed that SNW1 interacts with core spliceosome components NUDT21 and CPSF6 to modulate SRPK1 splicing. This study underscores the pivotal contribution of RNA splicing in BCa metastasis, with SNW1 serving as a key mediator. The identification of key regulatory molecules provides valuable insights into potential therapeutic targets for clinical intervention in metastatic BCa.

## Highlights

• SNW1 facilitates lymph node metastasis in bladder cancer through an SRPK1-dependent mechanism
• SNW1 modulates the splicing of SRPK1 by interacting with NUDT21/CPSF6
• Treatment with an SRPK1 inhibitor suppresses bladder cancer metastasis

## Graphical abstract

**Figure:**
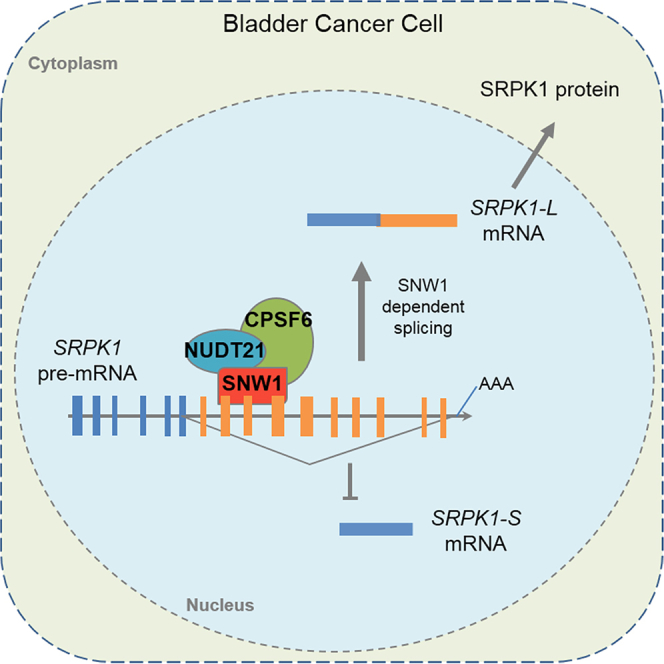

## Introduction

Metastatic bladder cancer (BCa) treatment poses significant challenges, with limited therapeutic options and poor patient outcomes.^1^ Recent advancements have introduced immune checkpoint inhibitors, targeted therapy, and antibody-conjugated drugs into the management of metastatic BCa; however, overall survival (OS) outcomes for patients remain suboptimal.^2^ Moreover, the selection of optimal therapies for patients with metastatic BCa continues to be hindered by the lack of reliable predictive biomarkers, such as tumor mutation burden, molecular subtypes, or gene expression profiles.^3^ A complex regulatory network in the tumor microenvironment cooperates to regulate metastasis and progression. Abnormal regulation of lncRNAs is associated with tumor metastasis by promoting immune escape.^4^ Excessive lactate accumulation in the tumor microenvironment is an important mechanism that promotes tumor invasion and metastasis.^5^ Lymphatic spread is the predominant mode of BCa, highlighting the urgent need to unravel the mechanisms driving lymph node (LN) metastasis.

Alternative splicing (AS) is a ubiquitous process in eukaryotic cells that is essential for modulating gene expression and enhancing proteomic complexity. Splicing dysregulation is an essential mechanism driving cancer metastasis and invasion.^6^ For instance, RNA splicing has been shown to regulate lung metastasis in breast cancer.^7^ PTBP1 enhances radioresistance in prostate cancer by regulating the alternative splicing of DNMT3B.^8^ Single-nucleotide polymorphisms (SNPs) associated with splicing are linked to an increased risk of BCa.^9^ The splicing factor NONO inhibits LN metastasis in BCa via splicing regulation.^10^ Despite these advances, the mechanisms through which RNA splicing participates in LN metastasis remain poorly understood. In this study, compared to adjacent normal tissues, the differentially expressed proteins in BCa tissues were most significantly enriched in the spliceosome pathway. Notably, SNW domain-containing protein 1 (SNW1) exhibited the most pronounced upregulation. Further analysis confirmed that elevated SNW1 expression was associated with LN metastasis and poor prognosis, suggesting its potential role in BCa metastasis.

SNW1, a member of the SNW gene family, is a highly conserved spliceosome component and transcriptional co-regulator. Its expression is elevated in various tumors and plays a role in cancer progression. For example, spliceosomes associated with SNW1 have been demonstrated to modulate apoptotic pathways in breast cancer cells.^11^ Furthermore, SNW1 regulates the expression of p21^Cip1^ through splicing, thereby counteracting p53-mediated apoptosis.^12^ In BCa, a previous study revealed that SNW1 is significantly upregulated in clinical tissues, and its elevated expression correlates closely with poor prognosis.^13^ However, the mechanism through which SNW1 regulates LN metastasis in BCa remains unclear.

This study revealed that SNW1 is conspicuously upregulated in BCa tissues and is significantly associated with LN metastasis and patient prognosis. SNW1 facilitates BCa metastasis by modulating the splicing of serine/arginine-rich protein-specific kinase 1 (SRPK1) through its interaction with nudix hydrolase 21 (NUDT21), and cleavage and polyadenylation specific factor 6 (CPSF6). These findings imply that the spliceosome pathway could be a key regulator of BCa metastasis.

## Results

### SNW domain-containing protein 1 is associated with lymph nodes metastasis in bladder cancer

**Figure 1:**
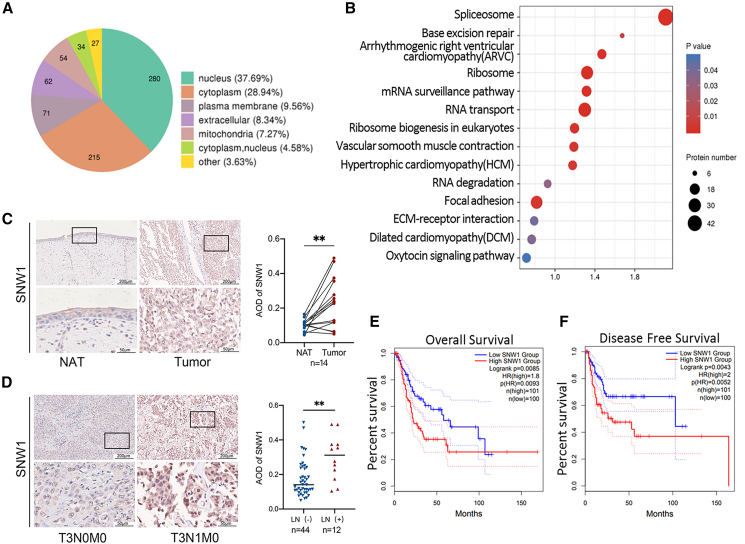
SNW1 expression correlates with LN metastasis and survival in bladder cancer (A) Label-free quantitative proteomics of cancer tissues (T) and paired adjacent normal tissues (N) from 17 patients with MIBC. Differentially expressed proteins (fold change >1.5, *p* < 0.05) were analyzed for subcellular localization. (B) KEGG pathway enrichment bubble plot of differentially expressed proteins. (C) Representative IHC staining of SNW1 in bladder cancer tissues (tumor) and normal adjacent tissues (NATs) from 14 patients. Quantitative evaluation of SNW1 expression represented as AOD. Data are presented as mean ± SD, ∗∗*p* < 0.01 (paired *t* test). Scale bars: upper panel = 200 μm, lower panel = 50 μm. (D) Representative IHC staining of SNW1 in bladder cancer tissues from patients with LN metastasis (*n* = 12) and without LN metastasis (*n* = 44). Quantitative evaluation of SNW1 expression represented as AOD. Data are presented as mean ± SD, ∗∗*p* < 0.01 (unpaired *t* test). Scale bars: upper panel = 200 μm, lower panel = 50 μm. (E and F) Kaplan-Meier survival curves for overall survival (E) and disease-free survival (F) in patients with bladder cancer stratified by low or high SNW1 expression (TCGA cohort).

To explore the mechanisms underlying BCa progression, we analyzed 17 pairs of muscle-invasive bladder cancer (MIBC) tissues (T) and adjacent normal tissues (N) using label-free quantitative mass spectrometry to measure protein signal abundance. Differential expression analysis and *p* value calculations were performed to identify the significantly altered proteins. In a previous study, we identified a set of differentially expressed proteins between BCa and adjacent normal tissues.^14^ Subcellular localization prediction and classification of these proteins indicated that the majority were localized in the nucleus (Figure 1A). KEGG pathway enrichment analysis and bubble plots were employed to display the significantly enriched pathways (Figure 1B). The enrichment of differentially expressed proteins was most significant in the spliceosome pathway, and 51 of the upregulated proteins were present in the spliceosome (Table S1).

In the spliceosome pathway, SNW1 exhibited a significant elevation in cancerous tissues. SNW1, a member of the SNW gene family, is a highly conserved spliceosome component and transcriptional co-regulator, underscoring its potential functional importance. Immunohistochemical (IHC) staining of BCa tissues and paired adjacent normal tissues confirmed these findings, revealing significantly elevated SNW1 expression in BCa samples (Figure 1C). Notably, SNW1 expression markedly increased in BCa cells with LN metastasis (Figure 1D). Database analysis further supported these findings, revealing significantly reduced OS and DFS in patients with BCa with high SNW1 expression (Figures 1E and 1F). Collectively, these findings suggest that high SNW1 expression correlates with LN metastasis and poor outcomes in BCa, establishing its role as a prognostic indicator and potential therapeutic target.

### SNW domain-containing protein 1 enhances the metastatic potential of bladder cancer cells

**Figure 2:**
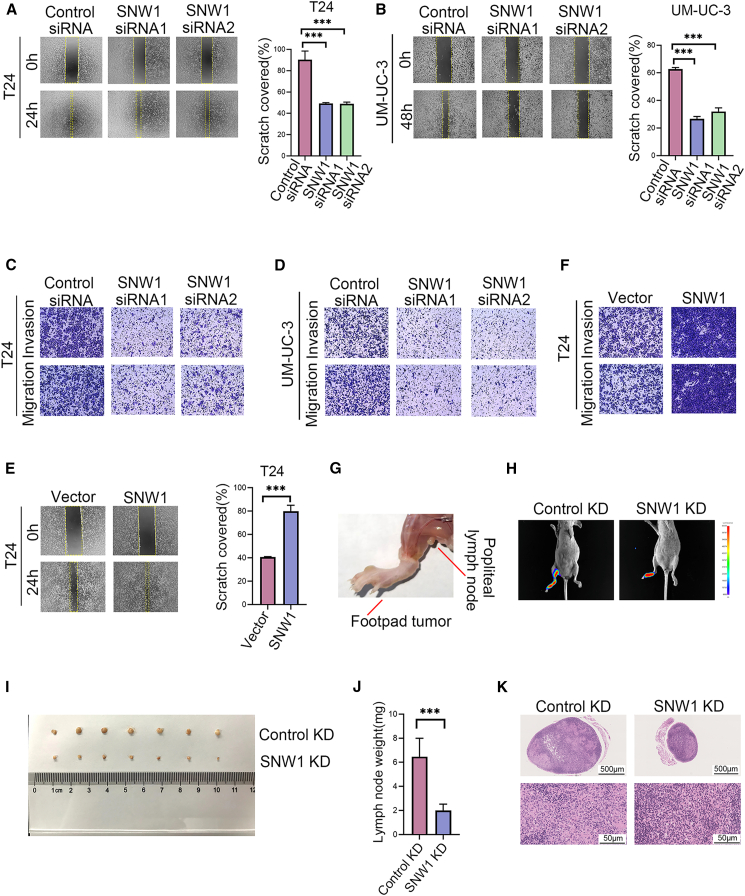
SNW1 promotes bladder cancer metastasis (A–D) Bladder cancer cells were transfected with SNW1-specific siRNAs or control siRNA. SNW1 knockdown significantly inhibited the migration of T24 (A) and UM-UC-3 (B) cells in wound healing assays, as well as their migratory and invasive potential in transwell assays (C, D). Data are presented as mean ± SD, ∗∗∗*p* < 0.001 (one-way ANOVA). (E and F) SNW1 overexpression enhanced T24 cell migration in wound healing assays (E), and increased migration and invasion in transwell assays (F). Data are presented as mean ± SD, ∗∗∗*p* < 0.001 (unpaired *t* test). (G) Schematic representation of the popliteal LN metastasis mouse model. (H) Representative bioluminescent images of LN metastasis in mice injected with SNW1 knockdown (SNW1 KD) or control knockdown (Control KD) T24 cells. (I and J) Representative images of dissected popliteal LNs (I), and quantitative analysis of LN weights (*n* = 7 per group) (J). Data are presented as mean ± SD, ∗∗∗*p* < 0.001 (unpaired *t* test). (K) HE staining of LNs from Control KD and SNW1 KD mice. Scale bars: upper panel = 500 μm, lower panel = 50 μm.

To confirm the function of SNW1 in cell migration and invasion, we employed RNA interference to knock down the expression of SNW1 (Figure S1A). Wound healing experiments revealed that SNW1 silencing markedly suppressed the migratory capacity of the cells (Figures 2A and 2B). Similarly, transwell migration and invasion experiments demonstrated that SNW1 knockdown substantially reduced the metastatic potential of the cells (Figures 2C and 2D). Conversely, the transient upregulation of SNW1 was achieved by transfection with an overexpression plasmid (Figure S1B), which enhanced the migratory and invasive properties of BCa cells (Figures 2E and 2F).

These data indicate that SNW1 is a key regulator of the metastatic capacity of BCa cells. To validate this *in vivo*, we generated a T24 cell line with stable SNW1 depletion and a matched control cell line (Figures S1C and S1D). The primary and most crucial initial site of BCa metastasis is the LN.^1^ BCa cells initially disseminate to pelvic LNs before spreading to distant organ sites.^15^ Hence, a popliteal LN metastasis model was selected to investigate metastatic processes *in vivo* (Figure 2G). The detection of luminescence intensity revealed that SNW1 knockdown significantly reduced metastasis to the popliteal LNs (Figure 2H). Consistent with this, the weight of popliteal LNs was lower in mice bearing SNW1 knockdown tumors than in the control group (Figures 2I and 2J). Furthermore, HE staining confirmed the presence of metastatic cancer cells in popliteal LNs (Figure 2K). These findings highlighted the involvement of SNW1 in LN metastasis during BCa progression.

### SNW domain-containing protein 1 regulates the RNA splicing of serine/arginine-rich protein-specific kinase 1

**Figure 3:**
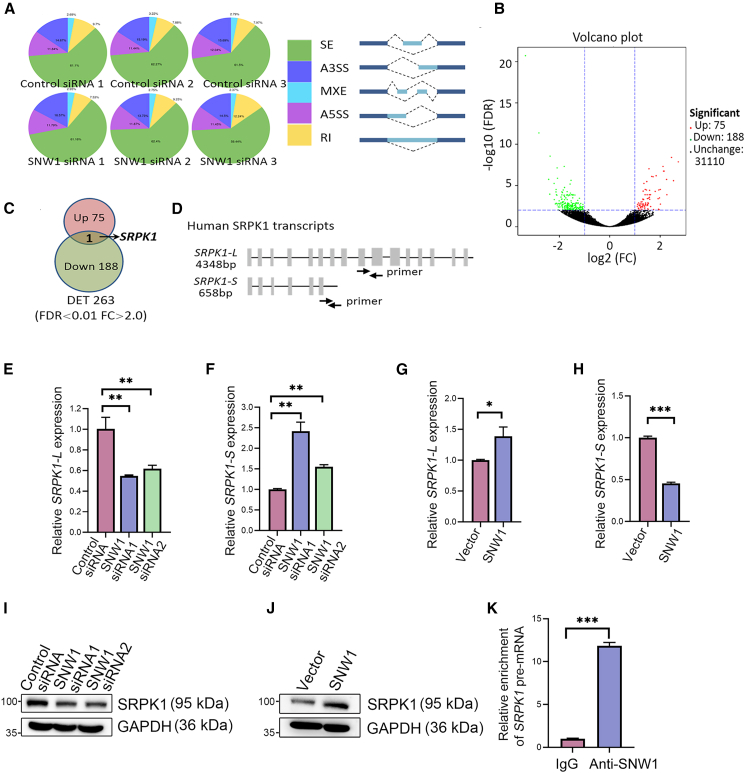
SNW1 regulates SRPK1 splicing (A) Quantification of AS events in T24 cells treated with three independent SNW1-targeting siRNAs. AS events were classified as skipped exon (SE), alternative 3′ splice site (A3SS), mutually exclusive exon (MXE), alternative 5′ splice site (A5SS), or retained intron (RI). (B) Volcano plot of differentially expressed transcripts following SNW1 knockdown (fold change ≥2, FDR <0.01). (C) Venn diagram shows overlap of upregulated and downregulated transcripts. (D) Schematic of SRPK1 transcripts shows primer pairs distinguishing the long (*SRPK1-L*) and short (*SRPK1-S*) isoforms. (E and F) RT-qPCR validation of *SRPK1-L* (E) and *SRPK1-S* (F) levels after SNW1 knockdown. Data are presented as mean ± SD, ∗∗*p* < 0.01 (one-way ANOVA). (G and H) RT-qPCR validation of *SRPK1-L* (G) and *SRPK1-S* (H) levels after SNW1 overexpression. Data are presented as mean ± SD, ∗*p* < 0.05, ∗∗∗*p* < 0.001 (unpaired *t* test). (I and J) Western blot analysis of SRPK1 protein levels following SNW1 knockdown (I) or SNW1 overexpression (J). (K) Quantification of *SRPK1* pre-mRNA enrichment in RIP assays using an anti-SNW1 antibody. RNA enrichment was normalized to IgG. Data are presented as mean ± SD, ∗∗∗*p* < 0.001 (unpaired *t* test).

To explore the AS events regulated by SNW1, we performed RNA interference experiments in T24 cells using three distinct siRNA sequences that specifically target SNW1. Nanopore full-length transcriptome sequencing was performed to distinguish between the different transcript isoforms. The outcomes demonstrated that Exon skipping was the most frequently observed AS event (Figure 3A). Differential transcript (DET) analysis revealed that 263 transcripts underwent significant alterations, including 75 upregulated and 188 downregulated isoforms (Figure 3B and Table S2). Among the transcripts affected by SNW1, *SRPK1* was uniquely identified and exhibited two diverse transcripts with opposing expression patterns (Figure 3C). Specifically, SNW1 knockdown markedly upregulated the shorter isoform *SRPK1-S* (encompassing exon 1–6), and decreased the full-length isoform *SRPK1-L* (encompassing exon 1–16) (Figure 3D). Full-length *SRPK1-L* (4348bp) is a known transcript that encodes the complete SRPK1 protein, whereas the shorter *SRPK1-S* (658bp) represents a novel transcript with unknown protein-coding potential and function.

SRPK1 is involved in mRNA splicing, export, translation, and other cellular processes by phosphorylating the serine/arginine-rich splicing factor 1 (SRSF1).^16^^,^^17^^,^^18^ Previous studies have indicated that SRPK1 enhances tumor metastasis via multiple pathways.^19^^,^^20^^,^^21^ However, the functional distinctions among different SRPK1 transcripts and their roles in BCa remain unclear. We performed RT-qPCR using transcript-specific primers that confirmed the presence of two distinct SRPK1 transcripts (Figure 3D). SNW1 knockdown significantly reduced the expression of *SRPK1-L* while elevating *SRPK1-S* levels (Figures 3E and 3F). Conversely, SNW1 overexpression increased *SRPK1-L* abundance and decreased the expression of *SRPK1-S* (Figures 3G and 3H). Changes in SRPK1 protein levels were also observed following SNW1 knockdown or overexpression (Figures 3I and 3J). To further investigate whether SNW1 directly regulates SRPK1 splicing, we conducted an RNA immunoprecipitation (RIP) assay, which demonstrated that SNW1 binds conspicuously to the intronic region of *SRPK1* pre-mRNA (Figure 3K). These results demonstrate that SNW1 directly modulates SRPK1 splicing and provide new insights into the molecular mechanisms underlying BCa progression.

### Serine/arginine-rich protein-specific kinase 1-L plays a critical role in promoting bladder cancer metastasis

**Figure 4:**
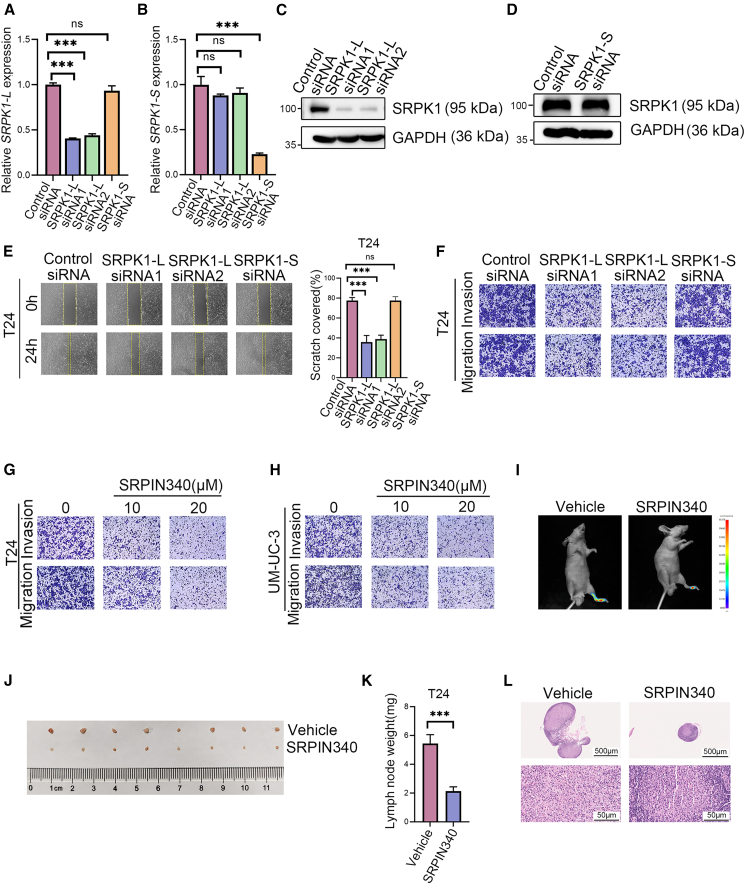
SRPK1-L plays a critical role in promoting BCa metastasis (A and B) RT-qPCR validation of *SRPK1-L* (A) and *SRPK1-S* (B) expression after isoform-specific siRNA knockdown. Data are presented as mean ± SD, ns, not significant, ∗∗∗*p* < 0.001 (one-way ANOVA). (C and D) Western blot analysis of SRPK1 protein levels following *SRPK1-L* (C) or *SRPK1-S* (D) knockdown. (E) Representative images and quantitative analysis of wound healing assays in T24 cells after *SRPK1-L* or *SRPK1-S* knockdown. Data are presented as mean ± SD, ns, not significant, ∗∗∗*p* < 0.001 (one-way ANOVA). (F) Representative images of transwell migration and invasion assays following *SRPK1-L* or *SRPK1-S* knockdown. (G and H) T24 (G) and UM-UC-3 (H) cells treated with SRPIN340 (0, 10, and 20 μM) for 48 h were assessed by transwell assays. (I) T24 cells are inoculated into the footpad of nude mice; representative bioluminescent images show LN metastasis in mice treated with SRPIN340 (0.8 mg/kg) or vehicle. (J) Representative images of dissected popliteal LNs. (K) Quantitative analysis of popliteal LNs weights (*n* = 8 per group). Data are presented as mean ± SD, ∗∗∗*p* < 0.001 (unpaired *t* test). (L) HE staining of LNs from SRPIN340 or vehicle-treated mice. Scale bars: upper panel = 500 μm, lower panel = 50 μm.

To clarify whether SRPK1 isoforms are involved in LN metastasis in BCa, we designed specific siRNAs targeting *SRPK1-L* or *SRPK1-S* transcripts. Knockdown specificity and efficiency were validated (Figures 4A and 4B). Notably, the specific knockdown of *SRPK1-L* led to a decrease in SRPK1 protein expression, whereas the knockdown of *SRPK1-S* had no significant impact on SRPK1 protein levels (Figures 4C and 4D). Knockdown of *SRPK1-L* significantly inhibited the migration and invasion of BCa cells, whereas the reduction of *SRPK1-S* expression had no significant effect on these cellular processes (Figures 4E, 4F, S2A, and S2B). These findings suggested that *SRPK1-L*, but not *SRPK1-S*, plays a critical role in promoting BCa metastasis, highlighting the functional divergence between the two isoforms.

SRPK1 inhibitors have been extensively investigated in preclinical studies. Our findings suggest that the SRPK1 inhibitor SRPIN340 can effectively suppress LN metastasis in BCa. Transwell experiments demonstrated that the SRPK1 inhibitor SRPIN340 repressed the migratory and invasive properties of BCa cells *in vitro* (Figures 4G and 4H). Consistent with the *in vitro* results, SRPIN340 treatment resulted in smaller popliteal LNs and fewer metastatic cancer cells (Figures 4I–4L). SRPK1 inhibitor SRPIN340 attenuates lymphatic metastasis of BCa cells *in vivo*.

### Serine/arginine-rich protein-specific kinase 1 is an effector that mediates the prometastatic function of SNW domain-containing protein 1

**Figure 5:**
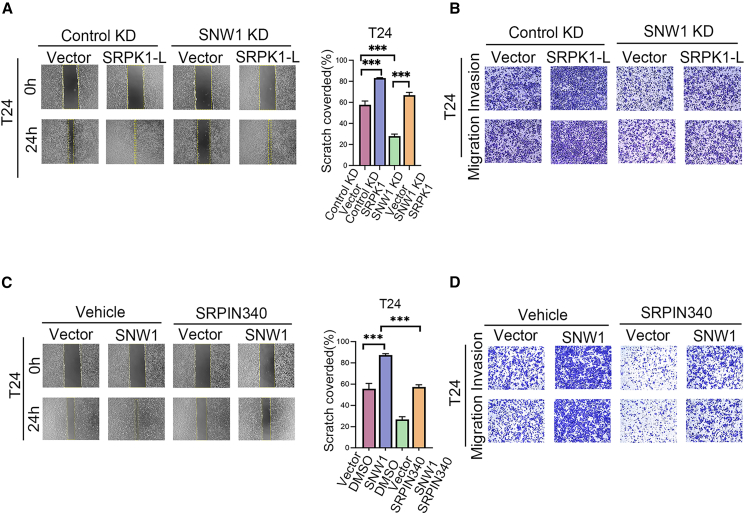
SRPK1 is an effector that mediates the prometastatic function of SNW1 (A) *SRPK1-L* overexpression or control vector was transfected into SNW1 KD and corresponding Control KD cells, and migration was assessed by wound healing assay. Data are presented as mean ± SD, ∗∗∗*p* < 0.001 (one-way ANOVA). (B) Transwell assays evaluating migration and invasion. (C) SNW1-overexpressing or control T24 cells were treated with SRPIN340 or vehicle, followed by wound healing assays. Data are presented as mean ± SD, ∗∗∗*p* < 0.001 (one-way ANOVA). (D) Migration and invasion were assessed using transwell assays.

To examine whether SNW1 facilitates LN metastasis in BCa by specifically regulating *SRPK1-L* transcript, we overexpressed *SRPK1-L* in SNW1-stably knockdown cells (Figure S1E). Interestingly, restoring *SRPK1-L* expression effectively rescued the motility and invasiveness of cancer cells, which were suppressed by SNW1 depletion. Additionally, overexpression of *SRPK1-L* alone significantly enhanced the metastatic potential of BCa cells (Figures 5A, 5B, S2C, and S2D). These data implied that SNW1 promoted LN metastasis in BCa through a mechanism dependent on *SRPK1-L* expression.

Moreover, SRPIN340 attenuated the enhanced migratory and invasive capabilities induced by the overexpression of SNW1 (Figures 5C, 5D, S2E, and S2F). These findings indicate that the SRPK1 inhibitor SRPIN340 can significantly suppress BCa metastasis and may have potential clinical applications in BCa treatment.

### SNW domain-containing protein 1 interacts with cleavage and polyadenylation specific factor 6/nudix hydrolase 21 to regulate the serine/arginine-rich protein-specific kinase 1 splicing

**Figure 6:**
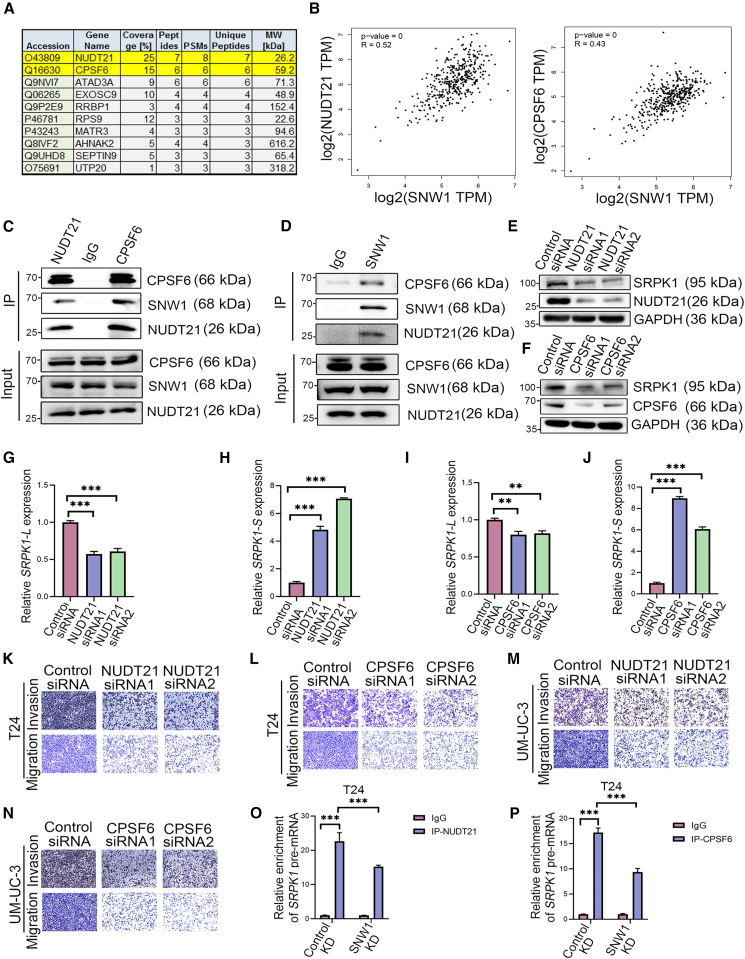
SNW1 interacts with NUDT21/CPSF6 to regulate SRPK1 splicing (A) Proteins interacting with SNW1 were immunoprecipitated with anti-SNW1 antibody and identified by LC‒MS/MS. Table lists the top 10 SNW1-associated proteins in T24 cells. (B) Pearson correlation analysis showing associations between SNW1 and NUDT21, and SNW1 and CPSF6, in the TCGA cohort. (C and D) CoIP validation of interactions between endogenous SNW1, NUDT21, and CPSF6. (E and F) Western blot analysis of SRPK1 protein following NUDT21 (E) or CPSF6 (F) knockdown. (G–J) RT-qPCR analysis of *SRPK1-L* (G, I) and *SRPK1-S* (H, J) transcript levels. Data are presented as mean ± SD, ∗∗*p* < 0.01, ∗∗∗*p* < 0.001 (one-way ANOVA). (K–N) Transwell assays show reduced migration and invasion of T24 (K, L) and UM-UC-3 (M, N) cells after NUDT21 or CPSF6 knockdown. (O and P) RIP assays with anti-NUDT21 (O) or anti-CPSF6 (P) antibodies showing reduced *SRPK1* pre-mRNA enrichment in SNW1 KD cells relative to Control KD cells. Data are presented as mean ± SD, ∗∗∗*p* < 0.001 (one-way ANOVA).

SNW1 participates in RNA splicing by interacting with splicing factors to form complexes.^22^^,^^23^ To ascertain whether SNW1 interacted with additional splicing factors, we performed co-immunoprecipitation (CoIP) employing an SNW1-specific antibody, followed by qualitative detection and analysis using protein mass spectrometry. Two candidate binding cleavage factors, nudix hydrolase 21 (NUDT21) and cleavage and polyadenylation specific factor 6 (CPSF6), were identified (Figure 6A). Interestingly, the analysis of the BCa data in GEPIA 2 revealed positive correlations between the expression levels of SNW1 and NUDT21, and between SNW1 and CPSF6 (Figure 6B). These interactions were further validated by coIP experiments, confirming the binding between SNW1 and NUDT21, as well as between SNW1 and CPSF6 (Figures 6C and 6D).

NUDT21 and CPSF6 can form heterodimers to manage the selection and processing of pre-mRNA cleavage and polyadenylation sites (PAS).^24^^,^^25^^,^^26^ To investigate the involvement of NUDT21/CPSF6 in *SRPK1* splicing, NUDT21 and CPSF6 were knocked down using specific siRNAs (Figures 6E and 6F). Knockdown of either NUDT21 or CPSF6 led to significant downregulation of the *SRPK1-L* isoform expression and concomitant upregulation of the *SRPK1-S* variant, consistent with the effects observed following SNW1 knockdown (Figures 6G–6J). These results suggest that SNW1 and NUDT21/CPSF6 collaboratively regulate *SRPK1* splicing by forming spliceosome complexes. Furthermore, the knockdown of NUDT21 or CPSF6 significantly inhibited the metastatic potential of BCa cells (Figures 6K–6N, Figure S3). RIP assays further confirmed that both NUDT21 and CPSF6 enriched the intronic fragments of *SRPK1* pre-mRNA. Interestingly, the binding of both NUDT21 and CPSF6 to *SRPK1* pre-mRNA was markedly inhibited in SNW1 KD cells (Figures 6O and 6P). Collectively, these results demonstrated that SNW1 interacts with NUDT21/CPSF6 to synergistically regulate *SRPK1* splicing, providing valuable mechanistic insights into BCa progression.

### SNW domain-containing protein 1 expression positively correlates with serine/arginine-rich protein-specific kinase 1 expression in bladder cancer

**Figure 7:**
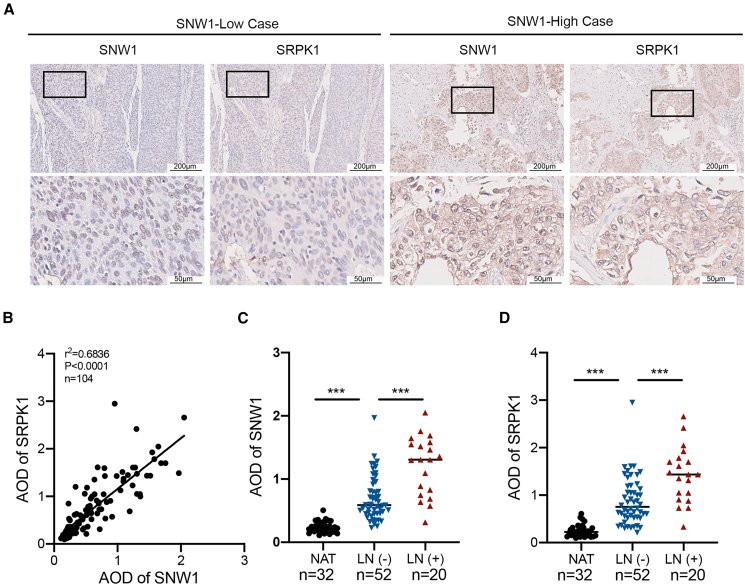
SNW1 positively correlates with SRPK1 expression and predicts LN metastasis in BCa (A) IHC staining of SNW1 and SRPK1 expression in BCa tissues. Scale bars: upper panel = 200 μm; lower panel = 50 μm. (B) Pearson correlation analysis of SNW1 and SRPK1 levels in 104 clinical bladder tissues (*r*^2^ = 0.6836, *p* < 0.0001). (C and D) AOD values of SNW1 (C) and SRPK1 (D) were compared across three tissue groups: normal adjacent tissues (NAT, *n* = 32), bladder cancer tissues without lymph node metastasis (LN−, *n* = 52), and with lymph node metastasis (LN+, *n* = 20). One-way ANOVA was performed to compare the differences among groups. Data are presented as mean ± SD, ∗∗∗*p* < 0.001.

To confirm the correlation and clinical significance of SNW1 and SRPK1, 72 BCa specimens and 32 normal adjacent tissue samples were collected. IHC analysis revealed that both SNW1 and SRPK1 were regionally upregulated in cancer tissues, and their expression levels were positively correlated (Figures 7A and 7B). Staining of two tissue sections from the same patient showed increased expression of both SNW1 and SRPK1 in cancer tissues compared to their normal counterparts (Figures 7C and 7D). Compared with cases without lymphatic spread, BCa tissues from patients with LN metastasis exhibited significantly elevated levels of SNW1 and SRPK1 (Figures 7C and 7D). These findings show a positive correlation between the expression of SNW1 and SRPK1, and both proteins are implicated in LN metastasis.

## Discussion

The clinical outcomes of patients with BCa with lymphatic metastasis remain unfavorable, with no significant improvement in prognosis. Therefore, elucidating the molecular basis of metastatic progression may provide novel preventive and therapeutic strategies. We identified SNW1, a splicing regulatory factor, as a key player in the progression of BCa. Elevated SNW1 expression was observed in tumor specimens and functionally contributed to the enhanced metastatic potential of BCa cells. Mechanistically, SNW1 facilitated BCa metastasis in an SRPK1-dependent manner by regulating the splicing of SRPK1 through its interaction with NUDT21 and CPSF6. Furthermore, the SRPK1-specific inhibitor SRPIN340 effectively suppressed BCa metastasis. This study revealed that an SNW1-associated splicing regulatory network is involved in BCa progression.

Important progress has been made in understanding the mechanism of LN metastasis in BCa, mainly focusing on molecular signaling pathways and tumor microenvironment regulation. A novel transcriptional program directed by the HSF1-PRMT5-WDR5 axis in the multistep process of lymphatic metastasis in BCa has been revealed.^27^ Targeting HSF1 may be an effective treatment strategy for patients with lymphatic BCa metastasis. The SLC2A11-MIF-PTBP1 axis was found to play an important role in the multistep progression of BCa by regulating mRNA stability.^28^ ETV4 promotes lymphangiogenesis and lymphatic metastasis by promoting the recruitment of BCa cells for tumor-associated neutrophil infiltration.^29^ The above mechanism provides new targets for the early blocking of lymphatic metastasis. An in-depth understanding of the mechanism of LN metastasis in BCa will help to develop precise treatment methods and improve patient prognosis.

Aberrant regulation of splicing can drive tumorigenesis and progression.^30^ However, the role of RNA splicing in the metastatic progression of BCa remains unknown. SNW1, an evolutionarily conserved protein in eukaryotes, primarily functions through protein-protein interactions.^31^ Structurally, SNW1 is divided into N-terminal, C-terminal, and middle SNW domains, featuring highly conserved regions that are essential for protein binding. These include a 5-amino acid SNWKN motif that is consistent across all species, and a conserved sequence adjacent to a predicted α-helix repeat. SNW1 exhibits structural flexibility and undergoes partial unfolding and refolding to facilitate dynamic protein interactions. As a spliceosome component, SNW1 directly interacts with multiple splicing factors and participates in pre-mRNA splicing and polyadenylation.^22^^,^^23^ Our investigation revealed that SNW1 interacts with the RNA splicing factors NUDT21 and CPSF6, which form heterodimers to regulate the selection and processing of pre-mRNA cleavage and polyadenylation sites (PASs).^24^^,^^25^^,^^26^ Although CPSF6 has not been studied in BCa, NUDT21 has been implicated in BCa progression; however, its target genes and molecular mechanisms remain elusive.^32^ Our data suggest that SNW1 modulates splicing through direct interactions with components of the splicing machinery, uncovering novel molecular mechanisms that contribute to BCa progression.

Our data indicate that SNW1 modulates SRPK1 splicing. SRPK1 plays a key role in post-transcriptional regulation, primarily by phosphorylating serine/arginine-rich splicing factor 1 (SRSF1), which alters cellular distribution and activity.^16^ SRSF1, a member of the SR protein family, is indispensable for RNA splicing, export, translation and other cellular processes.^17^^,^^18^ SRSF1 interacts with other spliceosome components to prevent exon skipping and ensure splicing accuracy.^33^ Given the critical role of spliceosomes in cancer, therapeutic strategies targeting this process, including SRPK1 inhibition, hold great promise for cancer treatment.^34^^,^^35^ SRPK1, in particular, represents a promising candidate for targeted therapeutic interventions and drug development. SRPK1 inhibitors have been used in preclinical studies. For example, SRPIN340 induces the expression of the anti-angiogenic isoform VEGF165b, thereby inhibiting angiogenesis.^36^ SRPIN340 also exhibits immunomodulatory activity, making it a potential candidate as a vaccine adjuvant or in immunotherapy.^37^ SRPKIN-1, another SRPK1 inhibitor, enhanced the expression of VEGF165b more effectively than SRPIN340.^38^ SPHINX31, a distinct SRPK1 inhibitor, triggers cell-cycle arrest and programmed cell death in acute myeloid leukemia (AML).^39^ In this study, SRPIN340 was found to inhibit LN metastasis in BCa cells, and these results were consistent with previous findings in other tumor types. Thus, SRPK1 and other key molecules within its regulatory network are promising molecular targets for BCa and warrant further comprehensive validation and exploration.

Splicing regulation is a pivotal mechanism that controls gene expression and proteomic complexity. More than 95% of human genes undergo AS, producing an average of approximately 10 transcripts per gene. AS can generate transcripts of the same gene with opposite functions. For example, the vascular endothelial growth factor (VEGF) promotes tumor angiogenesis. However, a novel VEGF isoform, VEGF165b, exhibited significantly reduced expression in malignant tissues compared to normal tissues. Studies have demonstrated that VEGF165b, an endogenous inhibitory form of VEGF, possesses antiangiogenic properties that counteract the proangiogenic effects of VEGF.^40^ Previous studies have shown that SRPK1 promotes tumor metastasis through diverse mechanisms. For instance, SRPK1 enhances pulmonary metastasis in breast carcinoma through NF-κB signaling.^19^ In colorectal cancer, SRPK1 regulates VEGF AS to promote angiogenesis.^20^ In contrast, targeting SRPK1 in prostate cancer induces the expression of the antiangiogenic isoform VEGF165b.^21^ However, whether SRPK1 promotes LN metastasis remains unclear. Moreover, previous studies have not distinguished between the different SRPK1 transcripts. Here, SNW1 knockdown inhibited *SRPK1-L* and reduced SRPK1 protein expression, while promoting the formation of a novel short transcript, *SRPK1-S*. Specific interference of the *SRPK1-L* transcript significantly inhibited the metastatic potential of BCa cells. AS is a potential therapeutic target.^41^^,^^42^ Neoantigens generated via splicing may offer novel strategies for cancer therapy.^43^^,^^44^ However, the functional role of the *SRPK1-S* transcript remains unknown and requires further investigation.

In summary, this study highlighted the crucial role of RNA splicing in BCa-LN metastasis, with SNW1 acting as an important mediator of SRPK1 splicing. The key splicing regulators identified in this study are potentially valuable targets and open new avenues for BCa intervention.

### Limitations of the study

This study has several limitations that should be noted. First, the detailed mechanism by which SNW1 promotes LN metastasis in BC cells remains to be elucidated. Second, the functional role of the *SRPK1-S* transcript warrants further investigation. Additionally, more research is needed to clarify the splicing mechanisms through which SRPK1 influences BC progression. While *in vitro* and *in vivo* models of BCa cells provide valuable simulations, they are unable to fully replicate the complex factors present in human tumors, such as the patient’s immune status, gender, and age.

## Resource availability

### Lead contact

Requests for further information and resources should be directed to and will be fulfilled by the lead contact, Wei Chen (chenw@njglyy.com).

### Materials availability

The unique materials generated in this study are available from the lead contact upon request.

### Data and code availability

• The raw sequence data have been deposited at Genome Sequence Archive (GSA) in National Genomics Data Center (NGDC), China National Center for Bioinformation (CNCB)/Beijing Institute of Genomics, Chinese Academy of Sciences, and are publicly available as of the date of publication. Accession numbers (GSA-Human: HRA013690) are listed in the key resources table.
• This article does not report original code.
• Any additional information required to reanalyze the data reported in this article is available from the lead contact upon request.

## Acknowledgments

This work was supported by grants from the 10.13039/501100001809National Natural Science Foundation of China (81972387, 82203765), Jiangsu Provincial Medical Key Discipline (Laboratory) Cultivation Unit (JSDW202221), Nanjing Health Distinguished Youth Fund (JQX20002).

## Author contributions

Conceptualization, W.C., H.G., and Q.Z.; methodology, J.H., Y.Z., G.W., H.D., H.H., W.C., M.D., W.D., and Q.L.; investigation, J.H., Y.Z., and H.D.; writing – original draft, J.H. and G.W.; writing – review and editing, W.C., H.G., and Q.Z.; funding acquisition, W.C., H.G., and Q.L.; all authors have read and approved the final article.

## Declaration of interests

The authors declare no competing interests.

## STAR★Methods

### Key resources table

**Table:** 

REAGENT or RESOURCE	SOURCE	IDENTIFIER
**Antibodies**		

SNW1	Bethyl Laboratories	Cat# A300-785A
SNW1	Proteintech	Cat# 25926-1-AP; RRID:AB_2880298
NUDT21	Proteintech	Cat# 10322-1-AP; RRID:AB_2251496
SRPK1	Proteintech	Cat# 14073-1-AP; RRID:AB_2194745
CPSF6	Proteintech	Cat# 15489-1-AP; RRID:AB_10694140
GAPDH	Proteintech	Cat# 60004-1-Ig; RRID:AB_2107436
Normal Rabbit IgG	Cell Signaling Technology	Cat# 2729; RRID:AB_1031062
HRP-Goat Anti-Rabbit Recombinant Secondary Antibody (H + L)	Proteintech	Cat# RGAR001; RRID:AB_3073505

**Bacterial and virus strains**		

hU6-MCS-CBh-gcGFP-IRES-puromycin	GeneChem	N/A

**Biological samples**		

Bladder cancer tissues and adjacent normal tissues	Nanjing Drum Tower Hospital, The Affiliated Hospital of Nanjing University Medical School	N/A

**Chemicals, peptides, and recombinant proteins**		

FBS	Gibco	Cat# A5256701
INTERFERin	Polyplus	Cat# 101000028
Lipomaster 2000	Vazyme	Cat# TL201
D-Luciferin, Potassium Salt	Vazyme	Cat# DD1210
Puromycin	Sangon Biotech	Cat# A610593
Pierce™ Protease Inhibitor	Thermo Scientific	Cat# A32955
Trizol reagent	Thermo Scientific	Cat# 15596018
Matrigel	Corning	Cat# 354234
4% paraformaldehyde	Biosharp	Cat# BL539A
Crystal Violet Staining Solution	Beyotime	Cat# C0121
Hematoxylin	Servicebio	Cat# G1004
Eosin	Servicebio	Cat# G1001
PureProteome Protein A/G magnetic beads	Millipore	Cat# LSKMAGAG10
PVDF membranes	Bio-Rad Laboratories	Cat# 1620177
SuperKine™ ECL	Abbkine	Cat# BMU102-CN

**Critical commercial assays**		

HiScript III RT SuperMix	Vazyme	Cat# R323
ChamQ Universal SYBR qPCR Master Mix	Vazyme	Cat# Q711
PureBinding®RNA Immunoprecipitation Kit	GENESEED	Cat# P0102

**Deposited data**		

Full-length transcriptome sequencing data	This paper	GSA-Human: HRA013690; https://ngdc.cncb.ac.cn/gsa-human/browse/HRA013690

**Experimental models: Cell lines**		

T24	Cell Bank of Chinese Academy of Sciences, Shanghai	Cat# TCHu 55
UM-UC-3	Cell Bank of Chinese Academy of Sciences, Shanghai	Cat# TCHu217

**Experimental models: Organisms/strains**		

BALB/c nude mice	GemPharmatech	N/A

**Oligonucleotides**		

See Table S3	Generay	N/A

**Recombinant DNA**		

pcDNA3.1-FLAG-SNW1	Youbio	N/A
pcDNA3.1-FLAG-SRPK1	Youbio	N/A

**Software and algorithms**		

Image-Pro Plus	Media Cybernetics	https://www.media-cybernetics.com/
GraphPad Prism 7	GraphPad Sofware	https://www.graphpad.com/

### Experimental model and study participant details

#### Cell lines and cell culture

UM-UC-3 and T24 (Cell Bank of Chinese Academy of Sciences, Shanghai) were incubated in Dulbecco’s Modified Eagle Medium (DMEM) and McCoy’s 5A medium, respectively. The cells were sent to a third-party institution (Guangzhou Cellcook Biotech Co., Ltd.) for STR profiling and mycoplasma testing. Both T24 and UM-UC-3 cell lines identities are clear and not contaminated with mycoplasma. Both complete media were supplemented with 10% (v/v) fetal bovine serum (FBS) (Gibco, USA), penicillin (100 U/ml) and streptomycin (100 U/ml). Specific siRNAs (Generay; sequences in Table S3) targeting SNW1, SRPK1, NUDT21, CPSF6 and control siRNA were transfected using INTERFERin (Polyplus, France). The SNW1 and SRPK1 overexpressing plasmids (Youbio, China) were delivered via Lipomaster 2000 (Vazyme, China). The cells exposed to Lentiviral shRNAs (GeneChem) were screened with purinomycin.

#### Animal experiments

Four-week-old male BALB/c nude mice were purchased from GemPharmatech (Nanjing, China). All animal procedures were approved by the Institutional Animal Care and Use Committee of Nanjing Drum Tower Hospital (No. 2024AE01102), and performed following ARRIVE guidelines. Mice were housed under specific pathogen-free conditions in individually ventilated cages and handled using aseptic techniques. To establish the popliteal LN metastasis model, T24 cells (4 × 10^6^ per mouse) with stable SNW1 depletion or control cells were injected into the footpad. After 30 days, metastatic progression was assessed using *in vivo* imaging. And then the mice were anesthetized with isoflurane and euthanized by cervical dislocation to collect popliteal LNs for weighed and photographed. Tissue specimens were analyzed by H&E.

#### Ethical approval

The bladder cancer tissues (*n* = 72) and normal adjacent tissues (NATs) (*n* = 32) for analysis were sourced from patients undergoing surgery from 2019 to 2025 at Nanjing Drum Tower Hospital. In this investigation, the samples utilized were the residual tissues following pathological examination. Throughout the study and upon the release of the findings, the personal information of patients was strictly safeguarded and not disclosed. Human bladder cancer samples were collected and investigated in line with the Declaration of Helsinki. The study was approved by the IRB of Nanjing Drum Tower Hospital of Nanjing University Medical School (No. 2018-015-01).

### Method details

#### Label-free quantitative proteomic analysis

The protein profiling data employed in this study were previously published.^14^ The concise process encompassed tissue protein extraction and digestion, high-performance liquid chromatography (HPLC) separation, liquid chromatography-tandem mass spectrometry (LC-MS/MS), and bioinformatics analysis. Proteins with a fold change >1.5 in expression and a *p*-value <0.05 were regarded as significantly differentially expressed. Kyoto Encyclopedia of Genes and Genomes (KEGG) database was used for pathway enrichment analysis. Fisher exact two-end test was used to examine the differentially expressed proteins.

#### Nanopore full-length transcriptome sequencing

Full-length transcriptome sequencing is conducted using Oxford Nanopore Technologies real-time sequencing technology. This technology enables the reading of sequence information encompassing a single complete transcript. The experimental procedures included sample quality detection, library construction, library quality detection and library sequencing. Three duplicate samples of SNW1 knockdown cells and their control cells were sequenced, and DESeq2 R package was used to analyze the differential expression between the two groups. Genes identified by DESeq2 with FDR <0.01 and foldchange ≥2.0 were designated as differentially expressed genes. The raw sequence data have been deposited in the Genome Sequence Archive^45^ in National Genomics Data Center,^46^ China National Center for Bioinformation/Beijing Institute of Genomics, Chinese Academy of Sciences (GSA-Human: HRA013690).

#### Western blotting (WB)

Cell lysates were extracted using cell lysis buffer supplemented with proteinase inhibitors. The proteins in each sample were separated by SDS-PAGE and subsequently transferred to PVDF membranes. Blots were blocked with 5% skim milk, then incubated with primary antibodies (Proteintech) at 4°C overnight. After washing, the membranes were treated with HRP-conjugated secondary antibodies for 1 h at RT. Primary antibodies against SNW1 (259261-AP), NUDT21 (10322-1-AP), SRPK1 (14073-1-AP), CPSF6 (15489-1-AP) were from Proteintech (Wuhan, China). Protein signals were detected by enhanced chemiluminescence (ECL) reagent, and imaged with ChemiScope 3300 Mini (CLiNX).

#### Real-time quantitative polymerase chain reaction (RT‒qPCR)

Total RNA was extracted with TRIzol reagent and reverse transcribed into cDNA using the PrimeScript RT kit. PCR systems were performed using ChamQ Universal SYBR qPCR Master Mix (Vazyme), and reactions were run on a QuantStudio 6 (Applied Biosystems, Foster City, USA). Relative mRNA expression levels were calculated using the 2^−ΔΔCT^ method. Primer sequences were listed in Table S3.

#### Cell migration and invasion assays

In the wound healing experiment, confluent cell monolayers were scratched using sterile pipette tips and cultured in serum-free medium. Migratory capacity was assessed by quantifying wound closure rates. For transwell assays, cells (1 × 10^5^ cells/well) were seeded in upper chambers (8 μm) to migrate. For invasion assays, matrigel-coated were used. After incubation, membranes were fixed, stained and imaged.

#### Immunoprecipitation (IP)

Protein extracts were prepared in ice-cold IP lysis buffer supplemented with protease and phosphatase inhibitors. For IP, lysates were incubated with specific antibodies (2 μg/sample) or control IgG (2 μg/sample) overnight at 4°C with gentle rotation. Protein A/G magnetic beads were then added and incubated for 2 h. After centrifugation and washing, the immunoprecipitated proteins pulled down by magnetic beads were separated. Eventually, bound proteins were eluded for subsequent mass spectrometry or WB.

#### RNA immunoprecipitation assay (RIP)

RIP was performed with the Purebinding RIP Kit (GENESEED, China) in accordance with the manufacturer’s protocol. Cells (10^7^ per sample) were lysed using RIP buffer through freeze-thaw treatment. Antibody-bound Protein A/G magnetic beads were prepared using primary antibodies (5 μg/sample) or control IgG overnight at 4°C. The antibody against SNW1 (A300-785A) was obtained from Bethyl Laboratories (USA). The immunoprecipitation complex was isolated, and the co-precipitated RNAs were eluted and extracted for subsequent RT-qPCR analysis.

#### Hematoxylin-eosin (H&E) and immunohistochemical (IHC) staining

Tissue specimens were preserved in 10% formalin at 4°C. Paraffin sections were incubated at 75° C, and processed through xylene and rehydrated through a graded ethanol series, and subjected to H&E staining using hematoxylin and eosin. For IHC, sections were blocked with 5% bovine serum albumin (BSA), then incubated with primary antibodies overnight at 4°C. After washing, HRP-conjugated secondary antibodies were applied for 1 h at RT. Signals were visualized using the DAB kit. Images were digitized using the NanoZoomer S60 (Hamamatsu).

### Quantification and statistical analysis

Data are presented as means ± standard deviation (SD). Statistical comparisons between two groups were determined by Student’s *t* test, whereas multiple group comparisons were analyzed by one-way ANOVA. All statistical computations were conducted using GraphPad Prism 7, with *p* < 0.05 considered statistically significant. Significant differences throughout all the figures are represented by ∗*p* < 0.05, ∗∗*p* < 0.01, ∗∗∗*p* < 0.001.
