# Supplementary material for: SNW1 promotes lymphatic metastasis in bladder cancer by modulating SRPK1 splicing
Source: iScience. 2026 Jan 27;29(2):114811. doi: 10.1016/j.isci.2026.114811 (PMC12907903; doi:10.1016/j.isci.2026.114811)
Supplement: Document S1. Figures S1–S3 and Tables S1–S3 [file mmc1.pdf]

iScience, Volume *29*

Supplemental information

SNW1 promotes lymphatic metastasis in bladder cancer by modulating SRPK1 splicing

Jijie Hu, Ying Zhu, Guoli Wang, Huiqi Dai, Haoyuan Han, Wenmin Cao, Meng Ding, Wenli Diao, Qun Lu, Qing Zhang, Hongqian Guo, and Wei Chen

Supplementary Figures

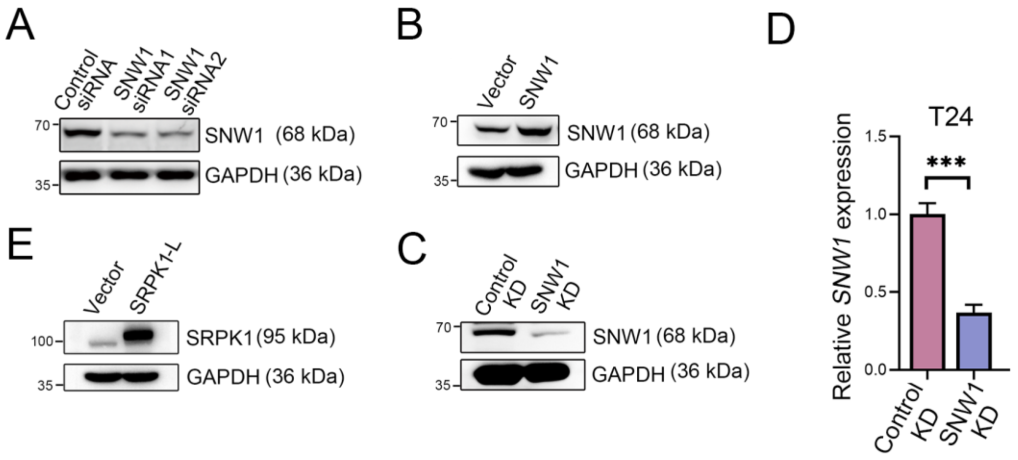

Figure S1. Knockdown and overexpression of SNW1. (A) Western blot analysis of SNW1 protein after siRNA knockdown in T24 cells. (B) Western blot confirming SNW1 overexpression in T24 cells. (C) Stable SNW1 knockdown (KD) generated using lentivirus; SNW1 protein detected by western blot. (D) SNW1 mRNA measured by RT-qPCR. Data are presented as mean ± SD, ***p < 0.001. (E) SRPK1 protein levels detected by western blot after *SRPK1-L* overexpression.

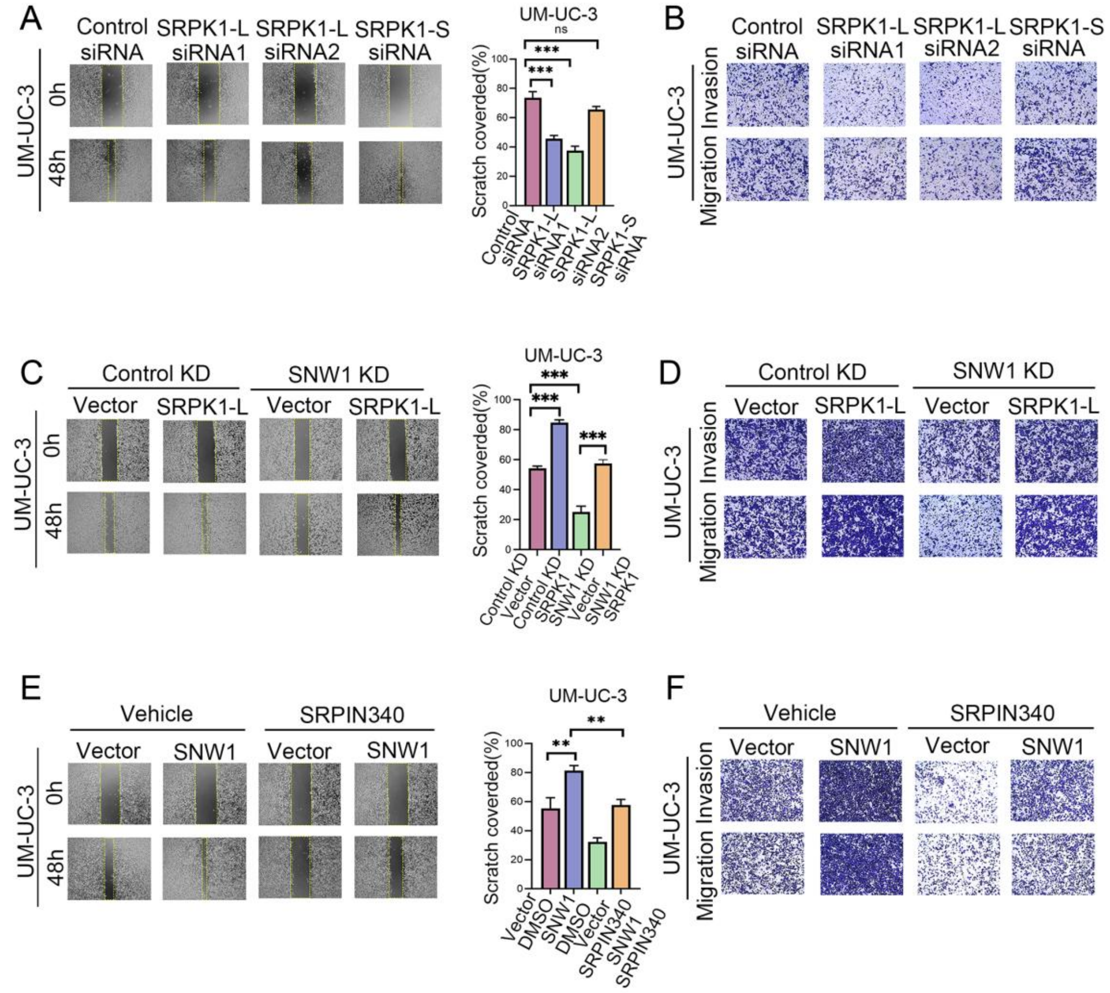

Figure S2. SRPK1-L promotes BCa cell metastasis. (A-B) Wound healing (A) and transwell assays (B) after *SRPK1-L* or *SRPK1-S* knockdown in UM-UC-3 cells. Data are presented as mean ± SD, ns, not significant, ***p < 0.001. (C-D) *SRPK1-L* overexpression or control vector transfected into SNW1 KD and Control KD UM-UC-3 cells; migration (C) and invasion (D) assessed. Data are presented as mean ± SD, ***p < 0.001. (E-F) UM-UC-3 cells transfected with SNW1 overexpression or control vectors and treated with SRPIN340 or vehicle; migration (E) and invasion (F) evaluated. Data are presented as mean ± SD, **p < 0.01.

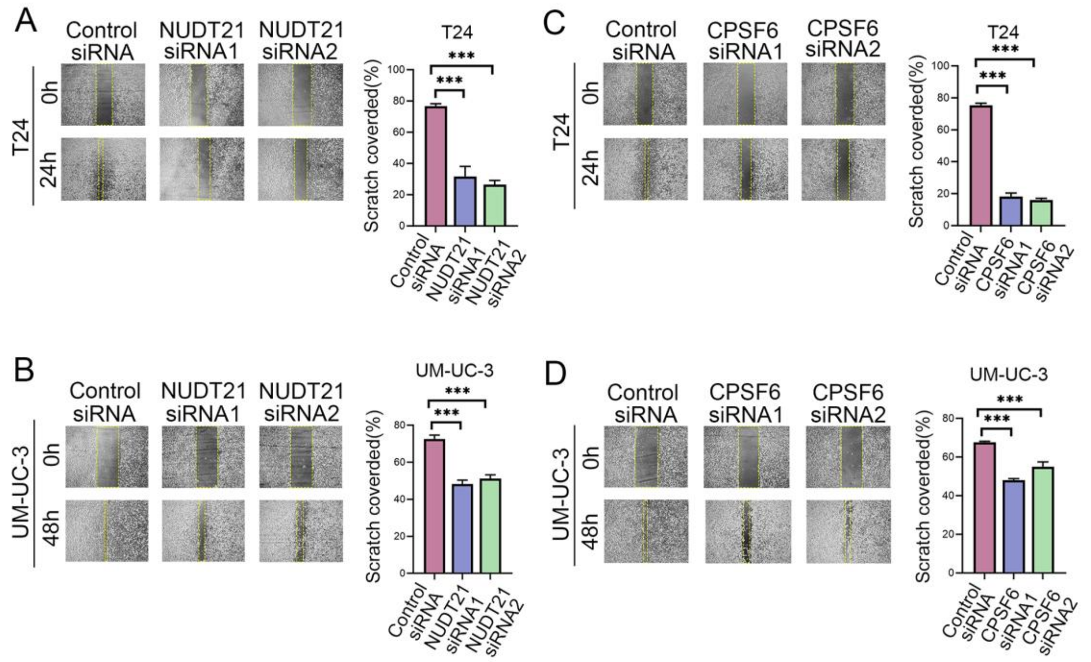

Figure S3. Knockdown of NUDT21/CPSF6 inhibits BCa cell metastasis. (A-B) Wound healing assays showing reduced migration after NUDT21 knockdown in T24 (A) and UM-UC-3 (B) cells. Data are presented as mean ± SD, ***p < 0.001. (C-D) Reduced migration after CPSF6 knockdown in T24 (C) and UM-UC-3 (D) cells. Data are presented as mean ± SD, ***p < 0.001.

Supplementary Tables

Table S1. Spliceosome associated proteins upregulated in bladder cancer tissues

Protein accession	Gene name	Mol. weight [kDa]	T.1/N.1 Ratio	T.1/N.1 P value	T.2/N.2 Ratio	T.2/N.2 P value
Q13573	SNW1	61.494	4.582	0.0018239	6.802	0.022745
O43290	SART1	90.254	4.846	0.0001021	5.7	0.0026968
Q96FV9	THOC1	75.665	2.424	0.0043352	4.698	0.0025811
Q8NI27	THOC2	182.77	4.415	0.0001414	4.549	0.0009771
Q8IWX8	CHERP	103.7	2.662	1.538E-05	4.215	0.0013398
Q99459	CDC5L	92.25	2.764	0.022555	3.865	0.0001649
Q9BUQ8	DDX23	95.581	2.78	0.0008447	3.784	0.0003845
O60306	AQR	171.29	2.87	2.06E-05	3.757	0.0014358
Q9Y2W2	WBP11	69.997	4.86	0.0010403	3.607	0.0004221
Q13247	SRSF6	39.586	3.799	0.0008611	3.473	0.0005182
O75400	PRPF40A	108.8	3.513	0.043897	3.288	0.0068763
Q13242	SRSF9	25.542	3.534	0.0026974	2.921	0.0007163
P09234	SNRPC	17.394	5.892	0.041825	2.891	0.0039615
Q9HCS7	XAB2	100.01	3.386	0.0015405	2.788	0.0097183
Q96I25	RBM17	44.961	2.521	0.032765	2.76	0.012996
O43395	PRPF3	77.528	3.501	0.0018822	2.711	0.0041572
O60231	DHX16	119.26	4.805	0.0036623	2.505	0.021721
P08579	SNRPB2	25.486	2.499	0.0076397	2.467	0.0004594
O43660	PLRG1	57.193	2.992	0.027543	2.435	8.343E-05
P49756	RBM25	100.18	3.769	0.0053367	2.307	0.0042801
O94906	PRPF6	106.92	2.415	0.0001448	2.281	0.0047983
P08621	SNRNP70	51.556	2.969	0.0102208	2.276	0.030235
P09661	SNRPA1	28.415	2.385	0.038918	2.274	0.0002976
P52272	HNRNPM	77.515	2.351	0.0126031	2.227	0.042223
P14678	SNRPB	24.61	3.01	0.0029792	2.218	0.0018846
O75533	SF3B1	145.83	2.591	0.023463	2.109	0.0152186
Q9BWJ5	SF3B5	10.135	2.618	3.646E-05	2.09	0.038341
Q9Y3B4	SF3B6	14.585	1.813	0.049116	2.071	0.031482
Q92620	DHX38	140.5	1.659	0.0015411	2.044	0.0136217
O75494	SRSF10	31.3	2.076	7.537E-05	2.035	0.035041
Q15428	SF3A2	49.255	3.154	0.0002198	2.034	0.039197
Q09161	NCBP1	91.838	2.395	0.006456	2.025	0.005581
Q8WYA6	CTNNBL1	65.173	2.572	0.0008841	2.016	0.0025011
P62306	SNRPF	9.7251	2.465	0.0001235	1.993	0.0034575
Q15029	EFTUD2	109.43	2.011	0.029704	1.948	0.0103796
O43143	DHX15	90.932	2.353	0.0155845	1.906	0.0053353
Q7RTV0	PHF5A	12.405	2.557	0.0061845	1.887	0.045784
O75934	BCAS2	26.131	2.003	0.0125618	1.88	0.030743

Q9Y5S9	RBM8A	19.889	2.621	0.0119577	1.868	0.0060039
Q15459	SF3A1	88.885	2.005	0.036325	1.853	0.039404
P83876	TXNL4A	16.786	1.598	0.0084448	1.841	0.0186036
O14776	TCERG1	123.9	2.042	0.0129026	1.828	0.0001821
Q00839	HNRNPU	90.583	2.167	0.0194825	1.817	0.0056982
P55769	SNU13	14.173	2.695	0.0067163	1.792	0.0141386
Q15393	SF3B3	135.58	1.953	0.048063	1.79	0.0080614
P09651	HNRNPA1	38.746	2.088	0.001344	1.788	0.0151166
P62314	SNRPD1	13.281	1.897	0.026375	1.78	0.0023598
P38919	EIF4A3	46.871	2.18	0.006221	1.761	0.0045436
P07910	HNRNPC	33.67	2.015	0.001681	1.673	0.0024049
Q9UHX1	PUF60	59.875	2.144	0.032664	1.634	0.028964
Q13838	DDX39B	48.991	1.928	0.047478	1.543	0.020059

Table S2. Differentially expressed transcripts after SNW1 knockdown

#ID	RNA_type	gene_name	Gene_symbol	FDR	log2FC
ENST00000553612	Known_mRNA	GTF2A1-203	GTF2A1	0.002759	-1.39847
ENST00000298310	Known_mRNA	NEMF-201	NEMF	0.000477	-1.91304
ENST00000261758	Known_mRNA	MESD-201	MESD	0.000522	1.346279
ENST00000371157	Known_mRNA	STAG2-204	STAG2	0.001221	-1.8747
ENST00000263461	Known_mRNA	WDR11-201	WDR11	0.004127	-1.74436
ENST00000392179	Known_mRNA	NDUFS2-202	NDUFS2	0.000947	1.281041
ENST00000374787	Known_mRNA	SMC2-202	SMC2	0.000134	-2.11786
ENST00000337130	Known_mRNA	UGP2-201	UGP2	0.004729	-1.28691
ENST00000473835	Known_mRNA	U2SURP-208	U2SURP	0.0038	-1.69803
ENST00000374293	Known_mRNA	GNG10-201	GNG10	0.002007	-1.0652
ONT.3322.1	New_mRNA	ONT.3322.1	SCG5	0.001269	-1.30999
ONT.8567.2	New_mRNA	ONT.8567.2	HIST1H2AC	0.008858	1.958631
ENST00000523916	Known_mRNA	CASP3-206	CASP3	0.001517	-1.46392
ONT.8269.3	New_mRNA	ONT.8269.3	SPDL1	5.69E-06	-2.20817
ENST00000440686	Known_mRNA	SLC26A10-202	SLC26A10	0.007792	-1.86556
ENST00000373825	Known_mRNA	SRPK1-204	SRPK1	0.003389	-1.15406
ENST00000554324	Known_mRNA	SNW1-203	SNW1	0.001535	-2.09853
ENST00000631619	Known_mRNA	CCNL1-226	CCNL1	0.009199	-1.67647
ONT.5757.11	New_mRNA	ONT.5757.11	EIF5B	0.000141	-1.90313
ENST00000428670	Known_mRNA	ATP2B1-204	ATP2B1	6.03E-05	-1.89945
ENST00000392594	Known_mRNA	THYN1-203	THYN1	0.001551	-1.33378
ENST00000261531	Known_mRNA	SNW1-201	SNW1	1.86E-21	-3.34679
ENST00000367993	Known_mRNA	NDUFS2-201	NDUFS2	0.003331	1.096087
ENST00000540933	Known_mRNA	GANAB-216	GANAB	0.001542	1.534557
ENST00000638108	Known_mRNA	IFIT2-203	IFIT2	0.000533	-1.7962
ENST00000333479	Known_mRNA	KNTC1-201	KNTC1	0.002338	-2.0798
ENST00000377392	Known_mRNA	CBWD5-204	CBWD5	0.003296	-1.51754

ENST00000489294	Known_mRNA	UHMK1-202	UHMK1	0.007387	-1.70933
ENST00000265433	Known_mRNA	NBN-201	NBN	0.006832	-1.50115
ENST00000475381	Known_mRNA	NCEH1-204	NCEH1	0.002759	-1.46825
ONT.2659.2	New_mRNA	ONT.2659.2	PHLDA1	0.000134	2.268773
ENST00000328333	Known_mRNA	COL7A1-201	COL7A1	0.004266	-1.19472
ONT.9620.3	New_mRNA	ONT.9620.3	TBC1D31	0.001604	-1.80476
ENST00000393561	Known_mRNA	LAMB1-204	LAMB1	0.007214	-1.10636
ENST00000314673	Known_mRNA	SNX14-201	SNX14	0.002744	-1.6817
ENST00000370008	Known_mRNA	STXBP3-201	STXBP3	0.002007	-1.8598
ENST00000649730	Known_mRNA	TFPI2-205	TFPI2	0.003996	1.378386
ENST00000370461	Known_mRNA	OGFR-202	OGFR	0.001866	1.4912
ENST00000444129	Known_mRNA	RECQL-204	RECQL	0.002455	-1.64151
ENST00000500692	Known_mRNA	GNPDA1-202	GNPDA1	0.0041	1.245087
ENST00000368400	Known_mRNA	DPM3-203	DPM3	0.001935	1.107561
ONT.6608.1	New_mRNA	ONT.6608.1	ONT.6608	0.002315	-1.88382
ENST00000454497	Known_mRNA	OTUD4-202	OTUD4	0.004122	-1.5062
ENST00000379391	Known_mRNA	NEMP1-202	NEMP1	0.002105	-1.49105
ENST00000283195	Known_mRNA	RANBP2-201	RANBP2	0.0041	-1.69781
ENST00000261173	Known_mRNA	ATP2B1-201	ATP2B1	1.83E-06	-2.42543
ENST00000341181	Known_mRNA	CLDND1-201	CLDND1	0.002322	-1.18995
ENST00000379883	Known_mRNA	DDX58-202	DDX58	1.36E-06	-2.07478
ONT.6536.2	New_mRNA	ONT.6536.2	SIRPB1	0.004931	-1.40879
ENST00000409600	Known_mRNA	BZW1-203	BZW1	0.00885	-1.14188
ENST00000379868	Known_mRNA	DDX58-201	DDX58	0.003996	-2.04171
ENST00000325805	Known_mRNA	BBX-201	BBX	0.003165	-1.78465
ENST00000395510	Known_mRNA	TWF1-201	TWF1	0.007626	-1.32836
ENST00000322244	Known_mRNA	UBA6-201	UBA6	0.001746	-1.68716
ENST00000263181	Known_mRNA	KIF18A-201	KIF18A	0.000947	-1.87156
ENST00000371065	Known_mRNA	LEPROT-201	LEPROT	0.007214	-1.17533
ONT.4755.3	New_mRNA	ONT.4755.3	PRPSAP1	1.83E-07	2.15829
ONT.3902.1	New_mRNA	ONT.3902.1	ONT.3902	0.000152	-2.10003
ENST00000359678	Known_mRNA	HIBCH-201	HIBCH	0.004812	-1.21248
ONT.7741.1	New_mRNA	ONT.7741.1	ONT.7741	0.005599	-1.63721
ENST00000346178	Known_mRNA	GANAB-201	GANAB	8.08E-07	1.667001
ENST00000376747	Known_mRNA	UGGT2-203	UGGT2	0.004976	-1.69487
ENST00000355451	Known_mRNA	NUDCD3-202	NUDCD3	0.001012	1.24458
ENST00000261191	Known_mRNA	INTS13-201	INTS13	0.002759	-1.56653
ENST00000434325	Known_mRNA	PLPP2-203	PLPP2	0.00185	2.012132
ENST00000356457	Known_mRNA	ASPH-201	ASPH	0.004266	-1.4852
ENST00000354185	Known_mRNA	DDX21-201	DDX21	0.005323	-1.32015
ENST00000521604	Known_mRNA	TCEA1-208	TCEA1	0.001517	-1.1519
ENST00000340344	Known_mRNA	NDUFV3-201	NDUFV3	0.007602	-1.07016
ENST00000412892	Known_mRNA	IGFN1-203	IGFN1	0.00438	-1.66642

ONT.6180.3	New_mRNA	ONT.6180.3	PAX8	0.00185	-1.48104
ENST00000202816	Known_mRNA	ESF1-201	ESF1	0.000973	-1.82925
ENST00000396946	Known_mRNA	CARD11-203	CARD11	0.003467	1.280086
ONT.9360.3	New_mRNA	ONT.9360.3	TFPI2	3.55E-05	1.948479
ENST00000454079	Known_mRNA	RBM6-213	RBM6	0.00469	-1.186
ENST00000378667	Known_mRNA	UQCRQ-202	UQCRQ	4.89E-05	1.326431
ONT.5680.1	New_mRNA	ONT.5680.1	ONT.5680	0.00022	-1.79368
ONT.6416.1	New_mRNA	ONT.6416.1	ONT.6416	0.006922	-1.79198
ENST00000354631	Known_mRNA	TRDMT1-202	TRDMT1	0.004064	-1.79948
ENST00000555761	Known_mRNA	SNW1-205	SNW1	4.27E-12	-2.81278
ENST00000355893	Known_mRNA	MLF1-201	MLF1	0.008171	-1.16289
ONT.3851.15	New_mRNA	ONT.3851.15	RPL13	0.009067	1.527103
ENST00000295830	Known_mRNA	RPL22L1-201	RPL22L1	3.55E-05	1.454394
ENST00000279477	Known_mRNA	SIRPB1-202	SIRPB1	5.68E-05	-1.69036
ENST00000542921	Known_mRNA	JMJD1C-210	JMJD1C	0.004064	-1.97854
ENST00000335211	Known_mRNA	IGFN1-202	IGFN1	7.56E-08	-2.08912
ENST00000332180	Known_mRNA	WASHC4-202	WASHC4	0.003677	-1.67316
ENST00000252816	Known_mRNA	LSM4-201	LSM4	0.00554	1.132262
ENST00000223127	Known_mRNA	PLOD3-201	PLOD3	0.00308	1.217604
ENST00000533723	Known_mRNA	TMEM41B-205	TMEM41B	1.86E-06	2.257667
ENST00000262210	Known_mRNA	CSPP1-201	CSPP1	0.002759	-1.75953
ONT.6336.1	New_mRNA	ONT.6336.1	ONT.6336	0.000736	-2.00895
ENST00000378292	Known_mRNA	TPM2-203	TPM2	9.69E-08	1.557635
ENST00000371122	Known_mRNA	SMARCA1-202	SMARCA1	0.008647	-1.47363
ENST00000216330	Known_mRNA	FKBP3-201	FKBP3	6.76E-06	-1.57442
ENST00000399107	Known_mRNA	POLR3G-201	POLR3G	3.11E-05	-2.13108
ONT.2790.1	New_mRNA	ONT.2790.1	ONT.2790	0.001535	-1.99415
ENST00000407559	Known_mRNA	WDR60-202	WDR60	0.006197	-1.42322
ONT.342.11	New_mRNA	ONT.342.11	UBAP2L	0.006792	1.387265
ENST00000309828	Known_mRNA	EIF3F-201	EIF3F	0.001744	1.24207
ENST00000316308	Known_mRNA	CLK4-201	CLK4	0.001517	-2.03039
ENST00000452853	Known_mRNA	PFN2-203	PFN2	0.003551	-1.05868
ONT.6535.3	New_mRNA	ONT.6535.3	SIRPB1	0.001606	-1.6213
ENST00000396679	Known_mRNA	CENPK-202	CENPK	0.001535	-1.41796
ENST00000296585	Known_mRNA	ITGA2-201	ITGA2	0.003947	-1.54204
ONT.9360.4	New_mRNA	ONT.9360.4	TFPI2	0.002455	1.408167
ENST00000356692	Known_mRNA	PPP4R2-201	PPP4R2	0.002824	-1.54757
ENST00000222553	Known_mRNA	NAMPT-201	NAMPT	0.00036	-1.2054
ENST00000530649	Known_mRNA	TAGLN-206	TAGLN	0.007602	1.939983
ENST00000367478	Known_mRNA	TPR-201	TPR	0.000256	-1.93497
ENST00000260641	Known_mRNA	ACTR2-201	ACTR2	0.003703	-1.07583
ENST00000569798	Known_mRNA	ALDOA-219	ALDOA	3.97E-09	2.451352
ONT.1992.1	New_mRNA	ONT.1992.1	AHNAK	2.66E-06	2.531603

ENST00000323666	Known_mRNA	METAP2-202	METAP2	0.007376	-1.32263
ONT.1521.8	New_mRNA	ONT.1521.8	RPLP2	0.00362	1.623191
ENST00000341119	Known_mRNA	SLC25A40-201	SLC25A40	0.008171	-1.53993
ENST00000436874	Known_mRNA	VEZT-204	VEZT	0.000339	-1.74287
ENST00000323981	Known_mRNA	TAF1D-201	TAF1D	0.005553	-1.33294
ENST00000358755	Known_mRNA	FZD6-201	FZD6	7.44E-05	-1.87842
ENST00000647435	Known_mRNA	TPM2-212	TPM2	0.001905	1.423811
ENST00000264279	Known_mRNA	NOP58-201	NOP58	0.000734	-1.53469
ENST00000498664	Known_mRNA	ATP6V0B-211	ATP6V0B	0.004122	1.303815
ENST00000263239	Known_mRNA	DDX18-201	DDX18	0.006338	-1.06973
ENST00000617677	Known_mRNA	EIF5B-205	EIF5B	3.55E-05	-1.84566
ONT.6391.2	New_mRNA	ONT.6391.2	TM9SF4	0.008403	1.742261
ONT.10563.1	New_mRNA	ONT.10563.1	PDZD11	0.000134	1.404166
ENST00000380062	Known_mRNA	IFT74-201	IFT74	0.004266	-1.55191
ENST00000307602	Known_mRNA	HOOK3-201	HOOK3	0.009929	-1.43403
ENST00000290271	Known_mRNA	STC1-201	STC1	0.000134	-1.66846
ENST00000507721	Known_mRNA	HNRNPDL-204	HNRNPDL	0.000175	-1.64894
ENST00000448108	Known_mRNA	TAF1D-203	TAF1D	0.008879	-1.13698
ONT.10400.2	New_mRNA	ONT.10400.2	OGT	0.001296	-1.76493
ONT.8852.8	New_mRNA	ONT.8852.8	SRPK1	3.11E-05	2.005116
ENST00000526600	Known_mRNA	TEAD1-203	TEAD1	0.0072	-1.5794
ENST00000248594	Known_mRNA	PTPN12-201	PTPN12	0.00133	-1.34419
ENST00000370982	Known_mRNA	GNG12-201	GNG12	0.0041	-1.19422
ENST00000359142	Known_mRNA	ATP2B1-202	ATP2B1	6.13E-05	-2.13468
ONT.2760.1	New_mRNA	ONT.2760.1	ONT.2760	8.57E-05	-1.56166
ENST00000354675	Known_mRNA	AKAP12-202	AKAP12	0.000134	-1.58897
ONT.7310.1	New_mRNA	ONT.7310.1	U2SURP	0.004485	-1.7943
ENST00000394144	Known_mRNA	TBC1D23-202	TBC1D23	0.006821	-1.68162
ENST00000370841	Known_mRNA	ACADM-202	ACADM	0.003154	-1.48644
ENST00000254654	Known_mRNA	ILKAP-201	ILKAP	0.002744	1.479259
ENST00000257247	Known_mRNA	AHNAK-201	AHNAK	0.004287	1.392117
ENST00000353245	Known_mRNA	YWHAZ-201	YWHAZ	0.004127	-1.12893
ENST00000372656	Known_mRNA	TCEAL9-201	TCEAL9	0.002905	-1.69339
ENST00000370952	Known_mRNA	LRRC40-201	LRRC40	0.002104	-1.68578
ONT.9339.1	New_mRNA	ONT.9339.1	ONT.9339	0.002954	-1.35371
ENST00000380629	Known_mRNA	BNIP3L-201	BNIP3L	0.004994	-1.08402
ENST00000264312	Known_mRNA	OCIAD1-201	OCIAD1	0.009924	-1.15214
ENST00000528419	Known_mRNA	CTSW-204	CTSW	0.001716	-1.93793
ENST00000289371	Known_mRNA	EIF5B-201	EIF5B	0.00133	-1.66698
ENST00000373669	Known_mRNA	PIN4-203	PIN4	0.009325	-1.05555
ENST00000300933	Known_mRNA	TPM4-201	TPM4	0.00027	1.575934
ENST00000217893	Known_mRNA	TAF9-201	TAF9	2.37E-06	-1.53672
ENST00000382722	Known_mRNA	CACNA2D4-202	CACNA2D4	0.001436	-2.12844

ENST00000265361	Known_mRNA	SEMA3C-201	SEMA3C	0.000318	-1.74429
ENST00000306061	Known_mRNA	MT1E-201	MT1E	0.000522	1.326732
ENST00000436063	Known_mRNA	DNTTIP2-202	DNTTIP2	0.00336	-1.34145
ENST00000379019	Known_mRNA	GPCPD1-201	GPCPD1	0.000522	-2.20364
ENST00000330439	Known_mRNA	MT1E-202	MT1E	0.007591	1.274661
ENST00000381719	Known_mRNA	FKBP1A-202	FKBP1A	5.01E-08	2.114391
ENST00000371648	Known_mRNA	EDF1-202	EDF1	0.000342	1.543707
ENST00000334651	Known_mRNA	DHRSX-201	DHRSX	0.000488	1.586771
ENST00000271452	Known_mRNA	NUF2-201	NUF2	0.000146	-1.71542
ENST00000532402	Known_mRNA	GANAB-211	GANAB	0.000134	1.57093
ENST00000397885	Known_mRNA	PUM3-202	PUM3	0.004213	-1.35437
ENST00000454859	Known_mRNA	ECHDC1-210	ECHDC1	0.007877	-1.35399
ENST00000611722	Known_mRNA	IFIT2-202	IFIT2	0.001939	-1.66777
ENST00000381055	Known_mRNA	ADAMTS6-203	ADAMTS6	0.005026	-1.87392
ENST00000366672	Known_mRNA	GALNT2-201	GALNT2	0.001186	1.518189
ENST00000245185	Known_mRNA	MT2A-201	MT2A	3.01E-05	1.408063
ENST00000478968	Known_mRNA	GLT8D1-206	GLT8D1	0.008075	-1.6497
ONT.4122.3	New_mRNA	ONT.4122.3	EMC6	3.39E-06	1.471457
ENST00000379463	Known_mRNA	NAE1-203	NAE1	0.005737	-1.25408
ENST00000290291	Known_mRNA	OGFR-201	OGFR	5.24E-05	1.804577
ENST00000343677	Known_mRNA	HIST1H1C-201	HIST1H1C	0.002315	1.515694
ENST00000396062	Known_mRNA	FKBP3-202	FKBP3	0.000146	-1.38465
ONT.4347.8	New_mRNA	ONT.4347.8	VMP1	0.000641	-1.27303
ENST00000298173	Known_mRNA	GTF2A1-201	GTF2A1	0.0072	-1.44533
ENST00000379731	Known_mRNA	B4GALT1-201	B4GALT1	0.001334	1.144438
ENST00000581216	Known_mRNA	TEFM-205	TEFM	0.009199	-1.5701
ONT.6054.1	New_mRNA	ONT.6054.1	COX7A2L	1.36E-08	2.723761
ENST00000398637	Known_mRNA	SLC38A1-201	SLC38A1	0.005697	-1.31519
ENST00000361804	Known_mRNA	SMC3-201	SMC3	0.007602	-1.49528
ENST00000278968	Known_mRNA	TAGLN-201	TAGLN	1.83E-07	2.135037
ENST00000306010	Known_mRNA	MGMT-201	MGMT	0.000442	1.31828
ENST00000372661	Known_mRNA	TCEAL9-202	TCEAL9	0.008422	-1.20314
ONT.10420.9	New_mRNA	ONT.10420.9	TCEAL9	0.002322	-1.60314
ENST00000397712	Known_mRNA	SEPT10-202	10-Sep	0.000149	-1.80981
ENST00000618348	Known_mRNA	GOLGA6L9-202	GOLGA6L9	0.001742	-2.15751
ONT.1221.1	New_mRNA	ONT.1221.1	CEP55	0.000134	-1.52267
ENST00000298299	Known_mRNA	ZNF22-201	ZNF22	0.001827	-1.36171
ENST00000621943	Known_mRNA	ELOVL1-216	ELOVL1	0.005188	1.219292
ONT.165.4	New_mRNA	ONT.165.4	ATP6V0B	0.002007	1.318679
ONT.5757.12	New_mRNA	ONT.5757.12	EIF5B	0.000442	-1.89592
ENST00000376389	Known_mRNA	FLOT1-201	FLOT1	0.001744	1.200628
ONT.10480.4	New_mRNA	ONT.10480.4	RPL10	3.91E-05	1.581486
ENST00000261475	Known_mRNA	PPP2R3C-201	PPP2R3C	0.003201	-1.46465

ENST00000371361	Known_mRNA	NOC3L-201	NOC3L	0.009067	-1.44822
ENST00000290810	Known_mRNA	NAE1-201	NAE1	0.006792	-1.22171
ENST00000319942	Known_mRNA	RABGGTB-201	RABGGTB	5.01E-08	-1.80823
ENST00000458500	Known_mRNA	RPL10-210	RPL10	0.002836	1.3571
ENST00000381605	Known_mRNA	SIRPB1-205	SIRPB1	0.000161	-1.58821
ENST00000263464	Known_mRNA	BIRC3-201	BIRC3	0.002631	-1.50228
ENST00000264977	Known_mRNA	PPP2R3A-201	PPP2R3A	0.006053	-1.6015
ENST00000265138	Known_mRNA	ARRDC3-201	ARRDC3	0.003537	-1.60315
ONT.268.3	New_mRNA	ONT.268.3	STXBP3	0.007645	-1.75801
ENST00000532359	Known_mRNA	RPL27A-211	RPL27A	0.001756	1.338438
ENST00000265112	Known_mRNA	TARS-201	TARS	0.001604	-1.26224
ENST00000370521	Known_mRNA	PKN2-203	PKN2	0.002982	-1.6532
ENST00000382456	Known_mRNA	AP2M1-202	AP2M1	0.007158	1.150376
ENST00000588734	Known_mRNA	TK1-203	TK1	6.64E-07	1.842478
ENST00000371485	Known_mRNA	CEP55-201	CEP55	0.002105	-1.36952
ENST00000418476	Known_mRNA	UNC45A-202	UNC45A	0.001334	1.422215
ENST00000345865	Known_mRNA	UPF3B-202	UPF3B	0.009067	-1.28977
ONT.9287.5	New_mRNA	ONT.9287.5	LSM5	0.000327	1.97429
ONT.2384.13	New_mRNA	ONT.2384.13	TXNRD1	0.009635	-1.20994
ENST00000613151	Known_mRNA	TPR-209	TPR	0.003524	-1.88893
ENST00000322954	Known_mRNA	UACA-201	UACA	0.004127	-1.52921
ENST00000219789	Known_mRNA	CDIPT-201	CDIPT	0.002901	1.309697
ENST00000355057	Known_mRNA	HIST1H4J-201	HIST1H4J	0.000404	1.91938
ENST00000281453	Known_mRNA	CENPU-201	CENPU	0.002414	-1.57204
ENST00000271469	Known_mRNA	UAP1-201	UAP1	0.000134	-1.48252
ENST00000378300	Known_mRNA	TPM2-204	TPM2	0.000134	1.485229
ONT.6703.1	New_mRNA	ONT.6703.1	ONT.6703	0.009635	1.201786
ENST00000460851	Known_mRNA	EIF2A-203	EIF2A	0.001221	-1.52592
ENST00000378743	Known_mRNA	ZFC3H1-201	ZFC3H1	0.002338	-2.00185
ONT.7650.6	New_mRNA	ONT.7650.6	CCNL1	0.001251	-1.93545
ENST00000228434	Known_mRNA	CD69-201	CD69	0.006102	-1.5632
ENST00000265295	Known_mRNA	SPDL1-201	SPDL1	2.83E-09	-2.36875
ENST00000398080	Known_mRNA	SCML1-204	SCML1	0.002677	-1.89865
ENST00000333003	Known_mRNA	NR2C1-202	NR2C1	0.007602	-1.59843
ENST00000202834	Known_mRNA	TMEM230-201	TMEM230	0.000134	-1.44803
ENST00000418646	Known_mRNA	GPCPD1-202	GPCPD1	0.004213	-2.02271
ENST00000544848	Known_mRNA	H2AFJ-203	H2AFJ	8.91E-07	1.72206
ONT.4348.1	New_mRNA	ONT.4348.1	ONT.4348	0.000318	-1.47035
ENST00000637790	Known_mRNA	ASAH1-258	ASAH1	0.006625	-1.46191
ONT.6535.8	New_mRNA	ONT.6535.8	SIRPB1	0.009924	-1.43759
ENST00000379341	Known_mRNA	PRDX4-202	PRDX4	5.69E-06	1.357065
ENST00000561491	Known_mRNA	MT2A-202	MT2A	0.002477	1.168349
ENST00000358746	Known_mRNA	TTC37-201	TTC37	0.005029	-1.67215

ENST00000371826	Known_mRNA	IFIT2-201	IFIT2	1.77E-05	-1.79596
ONT.1389.1	New_mRNA	ONT.1389.1	ONT.1389	0.007755	-1.53818
ENST00000399061	Known_mRNA	ZNF24-202	ZNF24	0.001604	-1.63088
ENST00000260970	Known_mRNA	PPIG-201	PPIG	0.000522	-1.68796
ENST00000397063	Known_mRNA	NFE2L2-202	NFE2L2	0.007158	-1.77636
ENST00000533626	Known_mRNA	EIF3F-208	EIF3F	0.002954	1.190287
ONT.6508.3	New_mRNA	ONT.6508.3	OGFR	0.000133	1.617875
ONT.5243.2	New_mRNA	ONT.5243.2	PRMT1	0.001754	1.302512
ENST00000379983	Known_mRNA	UACA-202	UACA	0.001517	-1.76729
ENST00000251636	Known_mRNA	DHX29-201	DHX29	0.003677	-1.49331
ENST00000224950	Known_mRNA	STN1-201	STN1	0.00133	-1.2524
ENST00000437150	Known_mRNA	GCA-204	GCA	0.004459	-1.36784
ONT.8953.1	New_mRNA	ONT.8953.1	ONT.8953	0.002824	-1.72755
ENST00000256108	Known_mRNA	IMPA1-201	IMPA1	0.008171	-1.1443
ENST00000504930	Known_mRNA	POLR3G-204	POLR3G	0.000115	-2.38271
ENST00000256897	Known_mRNA	CCNH-201	CCNH	0.000161	-1.46089
ONT.6883.1	New_mRNA	ONT.6883.1	ONT.6883	0.000156	-1.81941
ENST00000205402	Known_mRNA	DLD-201	DLD	0.005075	-1.15618
ENST00000319041	Known_mRNA	SH3BGRL3-202	SH3BGRL3	0.000393	1.497585
ENST00000271836	Known_mRNA	ADAM15-201	ADAM15	0.006731	1.513169
ONT.2514.1	New_mRNA	ONT.2514.1	ONT.2514	0.009199	-2.04472

Table S3. PCR primers and small interfering RNAs

**Sequences of primers used in this study**		
Genes	Forward(5'-3')	Reverse(5'-3')
SNW1	GCTCGACAAGGACAGTCAAAA	GCGGCGACCTTCTGTGATAC
SRPK1-L	CCAGCCTAAACCAGCTGACA	AATTTCTGCTGCACCACCCT
SRPK1-S	AAGTCAGTTCGCAATTCAGA	TTTCTTTGGGATTGGGATGA
SRPK1 pre-mRNA	ATCCCAATCCCAAAGAAAGG	TGCCTGAAAAGAGGACAAGAA
NUDT21	AGATTTCAGCGCATGAGGGAA	GCAGCAGTAACACATGGGGT
CPSF6	GGCGTGGACCACATAGACATT	CCATGTAATCTCGGTCTTCTGGG
**Sequences of siRNA used in this study**		
Genes	Forward(5'-3')	Reverse(5'-3')
SNW1 siRNA1	GGAGGUUAUGAAUGCAGAUTT	AUCUGCAUUCAUAACCUCCTT
SNW1 siRNA2	CCGAUGAAGAAGCUAUUAATT	UUAAUAGCUUCUUCAUCGGTT
SRPK1-L siRNA1	GAGAAUAGAUUGUAUCAAATT	UUUGAUACAAUCUAUUCUCTT
SRPK1-L siRNA2	GCAAUAGACUAGAACUGAATT	UUCAGUUCUAGUCUAUUGCTT
SRPK1-S siRNA	GAACUAGAUUUGUUGUGUATT	UACACAACAAAUCUAGUUCTT
NUDT21 siRNA1	GGACAGCUCUGUUGCAGCCAGAUUU	AAAUCUGGCUGCAACAGAGCUGUCC
NUDT21 siRNA2	UGAACCUCCUCAGUAUCCAUAUAUU	AAUAUAUGGAUACUGAGGAGGUUCA
CPSF6 siRNA1	GAGUAAAUGAUAUUUUGGAGA	UCCAAAAUAUCAUUUACUCCC
CPSF6 siRNA2	GAUAAAAUUUUUUGAAAAUCG	AUUUUCAAAAAAUUUUAUCUC
